# Two-years Postradiotherapy Biopsies: Lessons from MRC RT01 Trial

**DOI:** 10.1016/j.eururo.2017.12.017

**Published:** 2018-06

**Authors:** Antoine Kass-Iliyya, Gordana Jovic, Claire Murphy, Cyril Fisher, Isabel Syndikus, Chakiath Jose, Christopher D. Scrase, John D. Graham, David Nicol, Matthew R. Sydes, David Dearnaley

**Affiliations:** a MRC Clinical Trials Unit at UCL, London, UK; b North Bristol Trust, Bristol, UK; c Royal Marsden Hospital, London, UK; d Clatterbridge Centre for Oncology, Wirral, UK; e Department of Radiation Oncology, Auckland City Hospital, Auckland, New Zealand; f Ipswich Hospital, Ipswich, UK; g Taunton & Somerset National Health Service Foundation Trust, Musgrove Park Hospital, Taunton, UK; h Institute of Cancer Research and Royal Marsden Hospitals, Sutton and London, UK

**Keywords:** Prostate cancer, Biopsy, Conformal radiotherapy

## Abstract

**Background:**

The importance of 2-yr postradiotherapy prostate biopsy status remains uncertain.

**Objective:**

To assess the value of 2 year post treatment biopsies in a randomised trial of radiotherapy dose escalation.

**Design, setting, and participants:**

Between 1998 and 2001, 843 men with localised prostate cancer were randomised to receive either control-64 Gy or escalated-74 Gy conformal radiotherapy (CFRT) in the MRC RT01 trial in combination with 3–6-mo neoadjuvant androgen deprivation therapy. Prostate biopsies were planned at 2 yr from start of CFRT in suitable men.

**Outcome measurements and statistical analysis:**

Prostate biopsy results and prostate-specific antigen (PSA) levels performed at 2 yr post-CFRT were evaluated with long-term biochemical progression free survival (bPFS) and overall survival. Outcome measures were timed from the 2-yr biopsy using a landmark approach.

**Results and limitations:**

A 2-yr biopsy was performed in 312/843 patients. One hundred and seventy-seven patients were included in the per-protocol group with median follow-up of 7.8 yr from biopsy. Median PSA at biopsy was 0.5 ng/ml. Sixty-four bPFS events were reported: 46/145 (32%) in patients with negative, 6/18 (33%) suspicious, and 12/14 (86%) positive biopsies. A positive biopsy was prognostic of worse bPFS, going forward, compared with negative and suspicious biopsies, hazard ratio (HR) = 4.81 (95% confidence interval [CI]: 2.50–9.26, *p* < 0.001). The estimate for survival was HR = 1.58 (95% CI: 0.52–4.78, *p* = 0.42). PSA values at 2 yr between 1.01 ng/ml and 2.09 ng/ml were also associated with subsequent PSA failures (HR = 2.71, 95% CI: 1.98–3.71), bPFS events (HR = 2.45, 95% CI: 1.81–3.32), and prostate cancer-specific survival (HR = 2.87, 95% CI: 1.08–7.64) compared with PSA ≤1.0 ng/ml.

**Conclusions:**

Two-year postradiotherapy prostate biopsies have limited value in patients with PSA control but both positive biopsy and higher PSA status are strongly associated with future bPFS events. A policy of selected biopsy may provide an opportunity for early salvage interventions.

**Patient summary:**

Routine 2-yr postradiotherapy biopsy is not recommended but can be considered in selected patients with unfavourable post-treatment prostate-specific antigen levels who are suitable for early salvage treatments.

## Take Home Message

Two-year postradiotherapy biopsy results from a randomised controlled trial indicate that routine biopsy is not indicated but biopsy should be used selectively to guide local salvage treatments in patients with unfavourable post-treatment prostate-specific antigen results.

## 1 Introduction

There is controversy over the value of prostate biopsy after radiotherapy (RT) treatment for prostate cancer in predicting future survival and recurrence trends [1], [2]. The inherent difficulties in interpreting postradiation prostate biopsies [3] and debate regarding the optimal time of performing those biopsies have contributed to the uncertainty [1], [4].

Previous reports have suggested that men with a positive biopsy post-RT have a much worse prognosis than those with negative biopsies. However, most of these reports included a small number of patients, short-term follow-up, and heterogeneous methods of pathology reporting [5], [6], [7], [8]. Our study included a large, prospectively-recruited cohort of patients participating in a randomised controlled trial, with 10-yr follow-up and a single reference pathologist.

## 2 Materials and methods

### 2.1 Design of the RT01 trial and treatments

The design, objectives, patient eligibility criteria, treatment methods of the RT01 trial have been detailed previously [9], [10], [11]. In brief, consenting men with histologically confirmed T1b–T3a N0 M0 prostate cancer and prostate-specific antigen (PSA) levels <50 ng/ml were registered.

Patients having conformally-delivered radiotherapy were randomised to receive either a control schedule of 64 Gy/32f RT (Std-64 Gy) or an escalated schedule of 74 Gy/37f (Esc-74 Gy). Neo-adjuvant androgen deprivation therapy was administered 3–6 mo prior to RT and was maintained until the end of RT. Based on pathology grading, PSA, and T-stage, men were stratified into two groups according to the risk of seminal vesicles involvement (low or moderate/high risk) [12].

The trial followed the principles of the Declaration of Helsinki. Each centre attained ethical approval and participants gave separate informed consent for trial participation and the 2-yr biopsy.

### 2.2 Trial assessments

#### 2.2.1 Assessments

Before treatment each patient underwent a prostate biopsy, PSA measurement and local (digital rectal examination, transrectal ultrasound/magnetic resonance imaging [MRI]), lymph node (computed tomography [CT]/MRI), and metastases staging (bone scan, chest x-ray). PSA and digital rectal examination were performed post-RT (10 wk, 18 wk) and were repeated at 6 mo, 12 mo, 18 mo, and 24 mo, and annually thereafter. Full assessment of the disease was undertaken if there was clinical or biochemical evidence of disease recurrence, which included CT or MRI of the pelvis and bone scan. Prostate biopsy was performed 2 yr from start of RT in consenting patients without evidence of biochemical or clinical progression. This entailed transrectal two to four core biopsies or more if clinically indicated.

#### 2.2.2 Two-year biopsy review

The window for the 2-yr biopsy was determined retrospectively as 18–36 mo after starting RT. These biopsies were reviewed by local and central pathologists. The biopsy outcomes at central review were classified as: (1) positive, if haematoxylin and eosin staining showed evidence of residual malignancy, regardless of the scarcity of malignant cells, (b) negative, if no malignant cells were present, or (c) suspicious, if it was not possible to distinguish cancerous cells from radiation atypia, even after immunostaining (CK-34beta-E12) or PSA staining.

#### 2.2.3 Definition of biochemical failure and assessment of progression

Biochemical failure was considered to have occurred if both of two conditions were met in close accord with the Phoenix definition [10]: (1) PSA >2 ng/ml measured ≥6 mo after RT commenced and (2) rise in PSA from nadir level by ≥50%. Full re-evaluation of disease (CT/MRI/bone scan) was triggered if there was clinical or biochemical evidence of recurrence (trigger values: PSA ≥ 10 ng/ml and ≥50% of presenting PSA level) [13].

### 2.3 Outcome measures

The coprimary outcome measures in RT01 were survival and biochemical progression-free survival (bPFS). Survival was defined as time to death from any cause or censoring at date of last contact, bPFS as time to the first of: biochemical failure, death from prostate cancer, or development of local, nodal, metastatic disease, or date of last contact.

### 2.4 Analysis populations

#### 2.4.1 Per-protocol group

This is the main focus of this analysis. The per-protocol group (PPG) included only patients without a prior bPFS event who had 2-yr biopsy within the window and which was reviewed centrally. Exclusion criteria were: (1) bPFS event before or at biopsy and (2) biopsies performed outside the 18–36 mo window. Analyses were timed from the 2-yr biopsy.

#### 2.4.2 Local histopathology review 2-yr biopsy group

This included patients without a prior bPFS event who had 2-yr biopsy which was reviewed locally (with or without central review). The same exclusion criteria applied as for the PPG. Analyses were timed from the 2-yr biopsy.

#### 2.4.3 Exploratory group

This included only patients with a bPFS event at or before the 2-yr biopsy. Analyses were timed from randomisation.

#### 2.4.4 Two-year PSA group

This included all patients with a PSA value within 20–28 mo after randomisation (whether biopsied or unbiopsied), who were bPFS-event-free up to the point of the 2-yr PSA test, and with PSA <2 ng/ml at the time of the test. All outcome measures were timed from the date of 2-yr PSA test.

### 2.5 Statistical considerations

Kaplan-Meier plots and log-rank test were used to study the impact of the biopsy outcome on bPFS and survival. Cox models adjusted for seminal vesicle involvement risk group and allocated treatment were used to estimate the hazard ratio (HR).

Multivariate Cox proportional hazards models were applied to bPFS and overall survival, using backward selection. Covariates were kept in the final model if they were statistically significant at a level of *p* < 0.10. Multiple logistic regression was used to study the impact of covariates on the outcome of the biopsy. Negative and suspicious outcomes were grouped together since there was no evidence of a difference for each outcome measures using log-rank test.

Kappa statistic was used to determine agreement between the local and central review. Fisher's exact test was applied to test the association between the seminal vesicle (SV) involvement and outcome of the biopsy. All analyses used a two-sided 5% significance level.

## 3 Results

Data were frozen on August 2, 2011, matching the previous results paper [10].

### 3.1 Patient populations

Of the 843 men randomised in RT01, 312 men (37%) underwent a 2-yr biopsy of the prostate. Median time from starting RT to biopsy was 2.1 yr (interquartile range: 2.0–2.2). Three hundred and eleven out of 312 (99.7%) biopsies were reviewed locally and 223/312 (71%) were reviewed centrally.

**Fig. 1:**
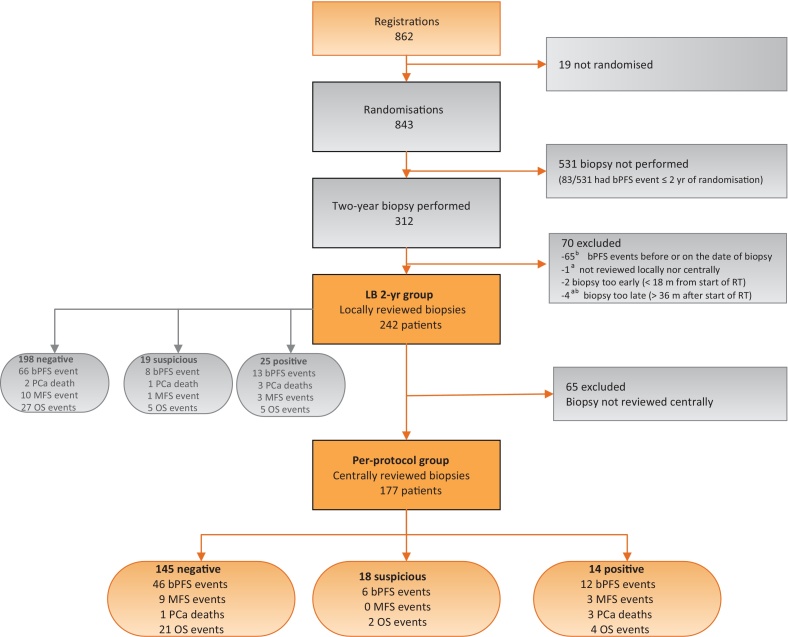
CONSORT diagram. bPFS = biochemical progression-free survival; LB = local histopathology review 2-yr biopsy; MFS = metastasis-free survival; OS = overall survival; PCa = prostate cancer; RT = radiotherapy. ^a^ 1 patient satisfies both criteria. ^b^ 1 patient satisfies both criteria.

Seventy out of 312 (22%) patients were excluded from the PPG, mainly (65/70) because a bPFS event was reported before or on the date of 2-yr biopsy (Fig. 1).

In the remaining 242 patients (local histopathology review 2-yr biopsy group [LBG]), biopsy outcomes were: negative 198/242 (82%), suspicious 19/242 (8%), and positive 25/242 (10%). The PPG consists of 177 patients whose biopsy outcomes were negative 145/177 (82%), suspicious 18/177 (10%), and positive 14/177 (8%). Kappa statistic showed good agreement between local and central pathologists assessments (kappa = 0.71; Table 1) [14].

**Table 1:** Agreement between local and reference histopathologist assessments

	Reference histopathologist				
Local histopathologist	Negative	Suspicious	Positive	Not done	Total
Negative	153	6	0	65	224
Suspicious	7	13	4	5	29
Positive	3	8	29	18	58
Not done	0	0	0	1	1
Total	163	27	33	89	312^a^

a Biopsy was performed in 312/843 patients.

### 3.2 PPG analyses (N = 177)

#### 3.2.1 Baseline characteristics

The median age, Gleason score and SV involvement risk groups of 177 patients in PPG were similar to the main trial population (Table 2).

**Table 2:** Baseline patient and tumour characteristics, by inclusion into per-protocol group (central review)

	Per-protocol group			All other patients^a^
	Negative	Suspicious	Positive	
	*N* (%)	*N* (%)	*N* (%)	
Age (yr)				
Median (IQR)	69 (65–72)	70 (66–72)	67 (63–71)	67 (63–71)
Mean	68	68	66	67
Range	47–80	58–76	51–74	47–81
T stage				
T1b–T2a	107 (74)	10 (56)	8 (57)	375 (57)
T2b	19 (13)	4 (56)	3 (21)	158 (24)
T3	19 (13)	4 (22)	3 (21)	121 (19)
Not known	0 (NA)	0 (NA)	0 (NA)	12 (NA)
Imputed Gleason score				
≤6	96 (66)	11 (91)	9 (64)	394 (60)
7	31 (21%0	7 (39)	4 (29)	180 (27)
≥8	18 (12)	0 (0)	1 (7)	87 (13)
Not known	0 (NA)	0 (NA)	0 (NA)	5 (NA)
PSA (ng/ml)				
Median (IQR)	10.0 (6.7–15.4)	11.6 (8.1–13.9)	15.0 (10.7–19.1)	13.4 (8.4–21.4)
Mean (SD)	12.7 (8.5)	13.5 (10.2)	15.0 (6.1)	16.0 (10.0)
Seminal vesicle risk group^b^				
Low	62 (43)	10 (56)	3 (21)	200 (30)
Moderate/high	83 (57)	8 (44)	11 (79)	466 (70)
Allocated treatment				
Std-64Gy	70 (48)	12 (67)	11 (79)	328 (49)
Esc-74Gy	75 (52)	6 (33)	3 (21)	338 (51)
Total	145	18	14	666

Esc-74 Gy = escalated 74 Gy; IQR = interquartile range; NA = not applicable; PSA = prostate-specific antigen; SD = standard deviation; Std-64 Gy = standard 64 Gy.

a Biopsy not performed (*N* = 531, patient did not meet the criteria for inclusion in per-protocol group (*N* = 70; Fig. 1), biopsy not reviewed by reference pathologist (*N* = 65).

b See Diaz et al [12].

A similar proportion of the Std-64 Gy group were included in the PPG biopsy cohort (93/421; 22%) as the Esc-74 Gy group (84/422; 20%) but biopsy-positive rates were lower in Esc–74 Gy (3/84; 4%) than Std-64 Gy (11/93; 12%).

In a multivariate analysis (logistic regression), considering age, T-stage, Gleason score, pretreatment PSA, SV involvement risk group and allocated treatment, the strongest associations with biopsy outcome were PSA value, as a continuous measure, at 2 yr (odds ratio [OR] = 1.90, 95% confidence interval [CI]: 0.97–3.71, *p* = 0.06), allocated treatment (OR = 0.31, 95% CI: 0.08–1.24, *p* = 0.10), and SV involvement risk group (OR = 2.61, 95% CI: 0.44–15.31, *p* = 0.29) but none of these reached statistical significance (Supplementary Table 1).

#### 3.2.2 bPFS

**Fig. 2:**
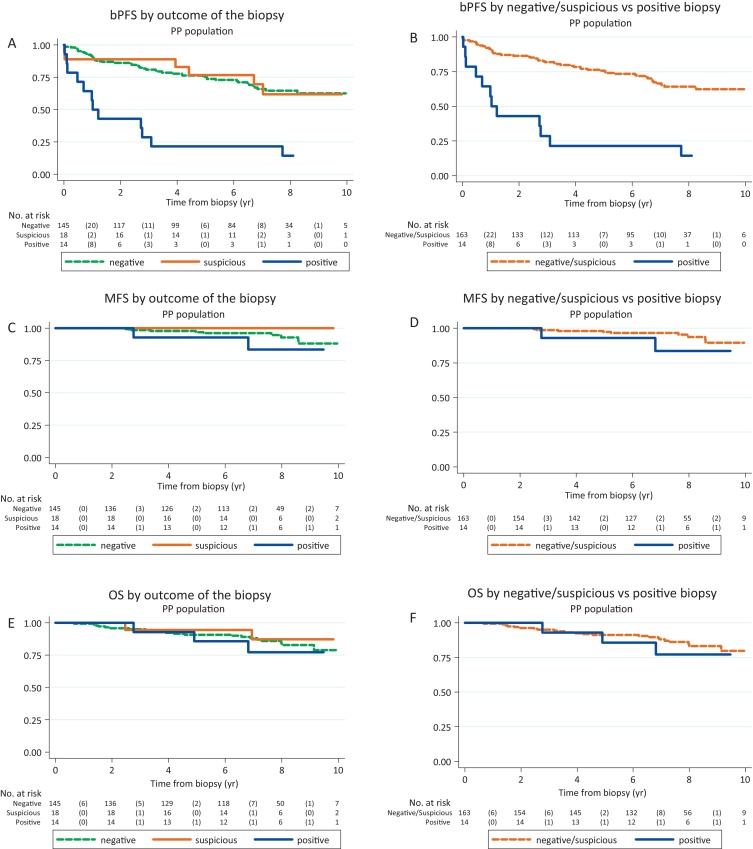
Outcome measures, per-protocol (PP) group (central review). (A) Biochemical progression-free survival (bPFS), by biopsy outcome. (B) bPFS, negative and suspicious versus positive biopsy. (C) Metastases-free survival (MFS), by biopsy outcome. (D) MFS, negative and suspicious versus positive biopsy. (E) Overall survival (OS), by biopsy outcome. (F) OS, negative and suspicious vs positive biopsy.

Sixty-four bPFS events were reported in the PPG: 46/145 (32%) in patients with negative, 6/18 (33%) suspicious and 12/14 (86%) positive biopsies, respectively. Patients with a positive biopsy were more likely to report a bPFS event in the future (HR = 4.81, 95% CI: 2.50–9.26, *p* < 0.001; Fig. 2A, Table 3).

**Table 3:** Outcome measures, per-protocol group (central review)

			Negative or suspicious biopsy	Positive biopsy
Outcome measure^a^	HR^b^ (95% CI)	*p* value	Events/patients	Events/patients
Biochemical progression-free survival	4.81 (2.50–9.26)	<0.001	52/163	12/14
Metastases-free survival	1.97 (0.49–7.92)	0.34	9/163	3/14
Overall survival	1.58 (0.52–4.78)	0.42	23/163	4/14
Prostate cancer deaths	15.64 (1.41–173.66)	0.02	1/163	3/14

CI = confidence interval; HR = hazard ratio.

a Timed from 2-yr biopsy.

b HR comparing “positive” versus “negative or suspicious” biopsy outcome, adjusted for seminal vesicle involvement risk group and allocated treatment.

The strongest associations with bPFS events on multivariate analysis were the outcome of the 2-yr biopsy (HR = 4.82, 95% CI: 2.53–9.19, *p* < 0.001), PSA at 2-yr (HR = 1.47, 95% CI: 1.20–1.81, *p* < 0.001), and ≥T3 stage (HR = 1.87, 95% CI: 1.03–3.40, *p* = 0.04).

Notably, 2/14 (14%) patients with positive prostate biopsies at 2-yr had no evidence of bPFS or clinical failure after completing 10-yr follow-up. Two-year PSA levels were 0.1 ng/ml and 0.2 ng/ml.

#### 3.2.3 Overall survival and prostate cancer-specific survival

Twenty-seven deaths were reported: 21/145 (14%) in patients with negative, 2/18 (11%) suspicious, and 4/14 (29%) positive biopsies, respectively (Figs. 2E and F2F). There was no statistically significant increased risk of death from any cause in patients with positive compared with suspicious or negative biopsies: HR = 1.58 (95% CI: 0.52–4.78, *p* = 0.42). Only 4/27 deaths were attributed to prostate cancer: 1/145 in patients with negative, 0/18 suspicious, and 3/14 positive biopsies, respectively: HR = 15.64 (95% CI: 1.41–173.66, *p* = 0.02).

### 3.3 LBG analyses (N = 242)

#### 3.3.1 Baseline characteristics

The median age, Gleason score, and SV involvement risk groups of the 311 LBG patients were similar to the main trial population (Supplementary Table 2).

#### 3.3.2 All outcome measures: results

Survival and bPFS results in the LBG group are broadly consistent with the PPG (Supplementary Table 3, Fig. 1), for example, for bPFS (HR = 1.64, 95% CI: 0.90–2.97, *p* < 0.001). Of the 37 deaths, only six (16%) were attributed to prostate cancer. Comparison of positive versus negative and suspicious biopsies in terms of prostate cancer-specific survival (PCSS) gave HR = 9.77 (95% CI: 1.61–59.20, *p* = 0.01). The strongest associations with bPFS were PSA ≥1 ng/ml at 2-yr (HR = 1.54, 95% CI: 1.30–1.83, *p* < 0.001) and ≥T3 stage (HR = 1.97, 95% CI: 1.17–3.33, *p* = 0.01).

### 3.4 Exploratory group (early failures, N = 65)

This consists of 65 patients who had a bPFS event before or on the date of their 2-yr prostate biopsy. Forty-six out of 65 of these biopsies were centrally reviewed. A higher proportion of these patients had a positive biopsy (19/46 [41%]) than the PPG (14/177 [13%]). There was an imbalance between the randomised groups with 32 (69%) of patients treated with 64 Gy and 13 (31%) treated with 74 Gy with biopsy positivity rates of 50% and 25%, respectively.

### 3.5 Overall death and prostate cancer-specific mortality comparing biopsied and nonbiopsied patients

The death rate was higher in the nonbiopsied patients; only 20.5% (64/312) of biopsied patients died compared with 32% (172/531) in the nonbiopsied group. Seventy patients were excluded from the LBG population, and 65/70 were excluded due to bPFS before 2 yr (the exploratory group [EG]). The death rate was much higher in this subgroup: 39% (27/70). Prostate cancer mortality was similar and low in the biopsied and unbiopsied populations who had PSA/clinical control at 2 yr with 6/242 (2%) and 19/448 (4%) deaths, respectively. However, it was considerably higher in patients who had PSA/clinical failure by 2 yr with 24% (17/70) and 59% (49/83) prostate cancer deaths in biopsied and unbiopsied cohorts, respectively (Supplementary Table 5).

### 3.6 Prognostic value of PSA at 2 yr

This can only be assessed in patients who had not previously reported a bPFS event and for whom a 2-yr PSA value was available: this was 621/843 (74%) patients.

#### 3.6.1 Two-year PSA and centrally-reviewed biopsies

Within these 621 patients, 2-yr biopsies had been performed in 251 (40%), with a central biopsy review in 179/251 (71%) patients. In these 179 patients, there was an association between higher 2-yr PSA values and positive biopsy (Fisher's test, *p* < 0.001); 11/31 (35%) patients with 2-yr PSA of 1.01–2 ng/ml had a positive biopsy on central review, compared with 10/148 (7%) with PSA <1 ng/ml. The median 2-yr PSA value was 0.5 ng/ml and, splitting at this point, positive biopsies were seen on central review for 9/95 (9%) patients with 2-yr PSA ≤0.5 ng/ml, 0/48 with PSA 0.51–0.99 ng/ml (0%) and 12/36 (33%) with PSA 1–2 ng/ml.

**Fig. 3:**
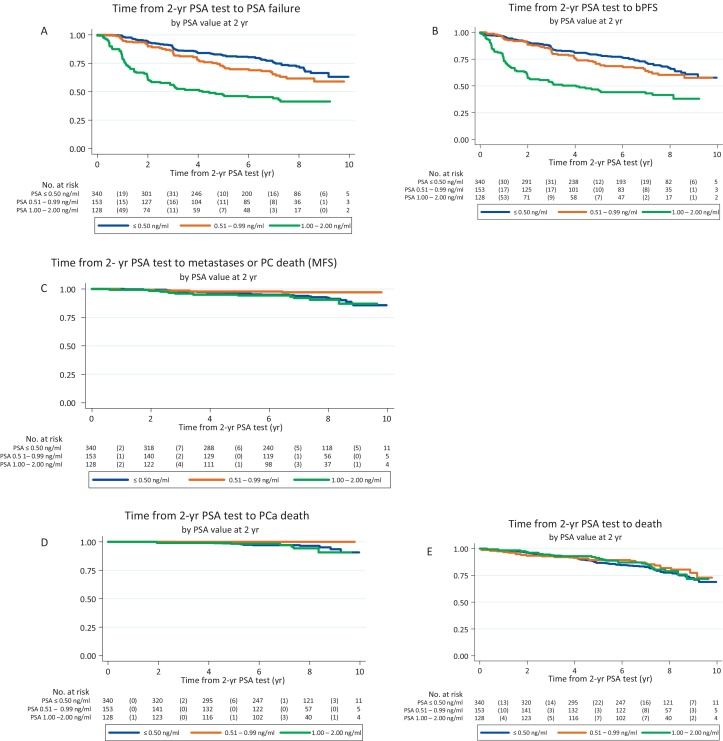
Time from 2-yr prostate-specific antigen (PSA) test to (A) PSA failure, (B) biochemical progression-free survival (bPFS) event, (c) metastasis-free survival (MFS) event, (D) prostate cancer (PC)-related death, (E) death; 2-yr PSA population.

Multivariate analyses in the 621 patients showed that 2-yr PSA was associated with subsequent PSA failures (HR = 2.71, 95% CI: 1.98–3.71), bPFS events (HR = 2.45, 95% CI: 1.81–3.32), PCSS (HR: 2.87, 95% CI: 1.08–7.64), but not clearly on metastasis-free survival (HR = 1.76, 95% CI: 0.86–3.60) or overall survival (HR = 0.95, 95% CI: 0.60, 1.51; Fig. 3, Supplementary Table 4).

## 4 Discussion

Failure to eradicate local tumours correlates with distant metastases development, cancer-related death, and overall survival [15], [16] and presently, post-RT prostate biopsy remains the only direct measure of local tumour control [17]. In agreement with other studies [4], [15], [16], [18], [19], our results show that prostate biopsies performed between 18 mo and 36 mo after RT are highly prognostic of future biochemical failure and disease–free status at 10 yr. As reported in previous studies, we found similar prognostic value for indeterminate and negative biopsies for subsequent biochemical failure [15], [17], [18], [20].

We recognise the low positive biopsy rate in our study [15], [17]. In particular, the differences between our study and RTOG 9408 which reported a 30% positive biopsy rate are of interest [19]. Potential causes for our lower biopsy positive rate include the use of dose escalation in 50% of the patients who had a considerably lower rate of positive biopsies (74 Gy 4% vs 64 Gy 12%), the use of 6 mo rather than 4 mo androgen suppression and the strict exclusion of patients who had PSA failure at the 2-yr time point. In our EG who had PSA failure before or on the date of their 2-yr biopsy, 41% had positive biopsies. Finally, our study used two to four biopsies and a higher positive biopsy rate might have been found if more comprehensive prostate sampling had been employed particularly if using MRI guidance.

We previously reported that 39% (91/236) of deaths in the RT01 trial were due to prostate cancer [10] and PCSS is a more appropriate outcome measure for assessing the value of positive prostate biopsies than overall survival. We observed significantly poorer PCSS in patients with positive 2-yr biopsies with HR of 15.6 and 9.8 in the PPG and LPG groups, respectively; however, our evidence is limited due to the small number of deaths (4/27 in PPG group), which probably relates to case selection for biopsy excluding patients with early PSA failure. Nevertheless, the data is in accord with Zelefsky et al [15] who demonstrated a three-fold increase in the rate of deaths from prostate cancer after 10-yr follow-up after a positive biopsy in intermediate- or high-risk prostate cancer patients treated with RT.

We noted that early biochemical failure correlates with a higher positive biopsy rate when compared with the general PPG; this could be attributed to early local recurrence reflecting more aggressive tumours. We also observed that biochemical failure does not always correlate with positive biopsies as 27/46 (59%) of cases with biochemical failure in the EG had negative biopsies. This is probably due to the development of extra-prostatic recurrence as the cause of PSA failure or, alternatively missing the recurrent focus of tumour during biopsy, yielding false-negative results.

PSA values at 2 yr were significantly correlated with biopsy outcomes, bPFS, PCSS, with PSA >1 ng/ml associated with a 2.7 higher chance of future biochemical failure than PSA ≤1 ng/ml. This is in agreement with previous studies reporting the importance of PSA nadir in predicting biochemical failure and disease-free survival [21], [22], [23], [24].

Prostate biopsy has disadvantages; it is an invasive procedure with risk of infection and bleeding. The uptake of biopsies in this study was modest with only 37% compliance. However, this is quite similar to the RTOG 9408 study where 42% of patients were biopsied [19] suggesting that this is the realistic proportion of patients who are likely to be suitable and consent to biopsy in such large multi-centre randomised trials. The timing of post-treatment biopsies is problematic and false-positive results occur. Crook et al [4] demonstrated that 30% of initially indeterminate biopsies at 13 mo cleared at a mean time of 31.6 mo; this is thought to be secondary to the prolonged killing effect of RT. In this series, two patients had positive biopsies at 2 yr with no evidence of biochemical recurrence 10 yr following RT. Conversely, sampling errors may lead to potential false-negative results.

Pathology interpretation is not straightforward and different assessment methods have been proposed [16], [21]. In this series, 27% (11/40) of biopsies scored positive by local pathologists were downgraded to indeterminate or negative.

Since this study multiparametric MRI (mpMRI) of the prostate has become an increasingly reliable method to diagnose loco-regional and distant recurrence combining T2 with diffusion weighted imaging [25], [26]. One recent study comparing mpMRI with histopathology after salvage prostatectomy post-RT showed 50–71% sensitivity with 80–100% specificity for detecting extra-prostatic extension [27]. A further study reported an area under the curve of 0.84 when using mpMRI to detect local recurrence compared with template transperineal biopsies [28]. Early results assessing prostate-specific membrane antigen-positron emission tomography appear promising [29].

Despite the association of positive post-RT prostate biopsies with future bPFS and CSS, we would not recommend routine biopsy in line with current practice in the UK. Biopsy remains essential in selected patients led by unfavourable post-treatment PSA profiles combined with MRI in patients suitable and favouring local salvage treatment. Clarification of the role of imaging and biopsy in patients with post-treatment PSA levels 1.0–2.0 ng/ml would be of value. For both the poor prognostic group of patients with PSA failure before 2 yr and an intermediate group with PSA levels 1–2 ng/ml at 2 yr imaging reassessment might be considered. Prostate biopsy would only be indicated after exclusion of extra-pelvic disease and there should be MRI or other imaging evidence of locally persistent disease. The patient must be suitable for and want local salvage treatment. For the good prognosis group of patients with PSA level of ≤1 ng/ml, the recurrence rate is low with excellent long-term outcomes and we would not recommend biopsy.

## 5 Conclusions

Prostate biopsies performed 2 yr after radical RT using contemporary doses with neoadjuvant androgen deprivation therapy are rarely positive in patients with PSA ≤2 ng/ml but are associated with poor outcome. PSA failure within 2 yr of RT identifies a population at high risk of death from prostate cancer. PSA-led prostate biopsies post-treatment should be considered in selected patients suitable for local salvage procedures.

  ***Author contributions:*** David Dearnaley had full access to all the data in the study and takes responsibility for the integrity of the data and the accuracy of the data analysis.  

*Study concept and design:* Kass-Iliyya, Fisher, Syndikus, Nicol, Sydes, Dearnaley.

*Acquisition of data:* Jovic, Murphy, Fisher, Syndikus, Jose, Scrase, Graham, Sydes, Dearnaley.

*Analysis and interpretation of data:* Kass-Iliyya, Jovic, Nicol, Sydes, Dearnaley.

*Drafting of the manuscript:* Kass-Iliyya, Jovic, Syndikus, Sydes, Dearnaley.

*Critical revision of the manuscript for important intellectual content:* Kass-Iliyya, Jovic, Murphy, Fisher, Syndikus, Jose, Scrase, Graham, Nicol, Sydes, Dearnaley.

*Statistical analysis:* Jovic, Murphy, Sydes.

*Obtaining funding:* Dearnaley, Sydes.

*Administrative, technical, or material support:* Murphy.

*Supervision:* Nicol, Dearnaley.

*Other:* None.  

***Financial disclosures:*** David Dearnaley certifies that all conflicts of interest, including specific financial interests and relationships and affiliations relevant to the subject matter or materials discussed in the manuscript (eg, employment/affiliation, grants or funding, consultancies, honoraria, stock ownership or options, expert testimony, royalties, or patents filed, received, or pending), are the following: None.  

***Funding/Support and role of the sponsor:*** The trial was sponsored by UK Medical Research Council and conducted by the MRC Clinical Trials Unit. Sydes, Jovic, and Murphy are employees of the sponsor at the Medical Research Council Clinical Trials Unit at UCL. MRC employees were central to the conduct of the trial and the development of this manuscript. Sydes and Jovic had access to raw data. The trial was registered on controlled-trials.com as ISRCTN47772397. Dearnaley, Nicol, Fisher, and Kass-Iliyya acknowledge NHS funding to the NIHR Biomedical Research Centre at the Royal Marsden NHS Foundation Trust and Institute of Cancer Research and Dearnaley has been supported by CRUK Program Awards C33589/A10588, C46/A10588, C46/A3976, C46/A2131.
