# Supplementary material for: Two-years Postradiotherapy Biopsies: Lessons from MRC RT01 Trial
Source: Eur Urol. 2018 Jun;73(6):968–76. doi: 10.1016/j.eururo.2017.12.017 (PMC5954168; doi:10.1016/j.eururo.2017.12.017)
Supplement: Supplementary file 1 [file mmc1.docx]

**Supplementary Table 1: Logistic regression, possible predictors for biopsy outcome*, PPG**

| **Possible predictor** | **Odds ratio (95%CI)** | **p-value** |
| --- | --- | --- |
| Age at randomisation | 0.95 (0.86-1.04) | 0.29 |
| Tstage |  |  |
| ≤T2a (reference) | 1.00 | n/a |
| T2b | 1.16 (0.23-5.75) | 0.23 |
| ≥T3 | 1.32 (0.26-6.78) | 0.74 |
| Gleason |  |  |
| ≤6 | 1.00 | n/a |
| 7 | 1.16 (0.26-5.05) | 0.85 |
| ≥8 | 0.59 (0.06-5.91) | 0.66 |
| Pre-hormone PSA | 1.00 (0.93-1.07) | 0.98 |
| Risk of seminal vesicle involvement |  |  |
| Low risk (reference) | 1.00 | n/a |
| Moderate risk | 2.61 (0.44-15.31) | 0.29 |
| Allocated treatment |  |  |
| Con-64Gy | 1.00 | n/a |
| Esc-74Gy | 0.31 (0.08-1.24) | 0.10 |
| PSA at 2 years | 1.90 (0.97-3.71) | 0.06 |

* negative and suspicious, or positive biopsy outcome

**Supplementary Table 2: Baseline patient and tumour characteristics, splitting biopsy groups**

|  | **Local histopathology review (LB) 2 year biopsy group** | | | **Exploratory group:**  **bPFS event before or on the date of 2-year biopsy** | **All other patients** |
| --- | --- | --- | --- | --- | --- |
|  | **Negative** | **Suspicious** | **Positive** |  |  |
|  | **N (%)** | **N (%)** | **N (%)** |  |  |
| **Age (years)** |  |  |  |  |  |
| Median  (IQR) | 69  (65-72) | 70  (61-72) | 70  (67-72) | 66  (61-70) | 68  (63-71) |
| Range | 47-80 | 51-79 | 54-76 | 47-78 | 47-81 |
| Mean | 68 | 67 | 69 | 65 | 67 |
| **T stage** |  |  |  |  |  |
| T1b - T2a | 141 (71%) | 11 (58%) | 16 (64%) | 30 (46%) | 302 (58%) |
| T2b | 29 (15%) | 6 (32%) | 5 (20%) | 20 (31%) | 124 (24%) |
| T3 | 28 (14%) | 2 (14%) | 4 (16%) | 15 (23%) | 98 (19%) |
| *Not known* | *0* | *0* | *0* | *0* | *12* |
| **Imputed Gleason score*** |  |  |  |  |  |
| ≤ 6 | 135 (68%) | 14 (74%) | 15 (60%) | 33 (51%) | 313 (59%) |
| 7 | 45 (23%) | 4 (21%) | 8 (32%) | 22 (34%) | 143 (27%) |
| ≥ 8 | 18 (9%) | 1 (9%) | 2 (8%) | 10 (15%) | 75 (14%) |
| *Not known* | *0* | *0* | *0* | *0* | *5* |
| **PSA (ng/ml)** |  |  |  |  |  |
| Median  (IQR) | 10.6  (6.8-16.3) | 11.7  (9.0-16.3) | 16  (10.2-19.2) | 16.7  (8.9-24) | 13.2  (8.4-21.4) |
| Mean  (SD) | 12.9  (8.8) | 13.1  (5.5) | 16.4  (10.3) | 18.7  (10.7) | 15.9  (9.9) |
| **Seminal vesicle**  **risk group** |  |  |  |  |  |
| Low | 88 (44%) | 9 (47%) | 8 (32%) | 13 (20%) | 157 (29%) |
| Moderate/high | 110 (56%) | 10 (53%) | 17 (68%) | 52 (80%) | 379 (71%) |
| **Allocated treatment** |  |  |  |  |  |
| Std-64Gy | 97 (49%) | 13 (68%) | 17 (68%) | 45 (69%) | 249 (46%) |
| Esc-74Gy | 101 (51%) | 6 (32%) | 8 (32%) | 20 (31%) | 287 (54%) |
| **Total** | **198** | **19** | **25** | **65** | **536** |
|  | **N=242** | | | **N=65** | **N=536** |

***** If Gleason score was missing, WHO differentiation was used in the following way: well, moderate, or poor differentiation is classified as Gleason score of 6, 7, or 8, respectively.

**Supplementary Table 3: Outcome measures, LB group (local review)**

|  |  |  | **Negative or**  **suspicious biopsy** | **Positive biopsy** |
| --- | --- | --- | --- | --- |
| **Outcome measure*** | **HR**^¥^ **(95%CI)** | **p-value** | **Events/pts** | **Events/pts** |
| Biochemical  Progression-Free Survival | 1.64 (0.90-2.97) | 0.10 | 74/217 | 13/25 |
| Metastases-Free Survival | 2.16 (0.59-7.91) | 0.25 | 11/217 | 3/25 |
| Overall Survival | 1.27 (0.49-3.28) | 0.62 | 32/217 | 5/25 |
| PCa death | 9.77 (1.61-59.20) | 0.01 | 3/217 | 3/25 |

*timed from 2-year biopsy

^¥^ HR comparing “positive” vs “negative or suspicious” biopsy outcome, adjusted for seminal vesicle involvement risk group and allocated treatment

**Supplementary Table 4: Multivariate models for all outcome measures^*^ in two-year PSA population**

|  | **Biochemical (PSA) failure** | **Biochemical Progression-Free Survival** | **Metastases-Free Survival** | **Prostate Cancer Specific Survival** | **Overall Survival** |
| --- | --- | --- | --- | --- | --- |
| **Variable** | **HR**^¥^ **(95%CI)**  **p-value** | **HR**^¥^ **(95%CI)**  **p-value** | **HR**^¥^ **(95%CI)**  **p-value** | **HR**^¥^ **(95%CI)**  **p-value** | **HR**^¥^ **(95%CI)**  **p-value** |
| **PSA value at 2 years after randomisation** |  |  |  |  |  |
| ≤0.5ng/ml | 1.00 (reference) | 1.00 (reference) | 1.00 (reference) | 1.00 (reference) | 1.00 (reference) |
| 0.51-0.99ng/ml | 1.35 (0.94-1.93)  p=0.101 | 1.18 (0.84-1.67)  p<0.001 | 0.35 (0.12-1.01)  p=0.051 | n/a (no PCa deaths in this group) | 0.79 (0.50-1.24)  p=0.304 |
| 1.00-2.00ng/ml | 3.10 (2.20-4.36)  p<0.001 | 2.61 (1.88-3.62)  p<0.001 | 1.32 (0.64-2.74)  p=0.455 | 1.62 (0.59-4.41)  p=0.348 | 0.88 (0.55-1.41)  p=0.604 |
| **Risk of seminal vesicle involvement** |  |  |  |  |  |
| Low | 1.00 (reference) | 1.00 (reference) | 1.00 (reference) | 1.00 (reference) | 1.00 (reference) |
| Moderate/high | 1.56 (1.15-2.12)  p=0.004 | 1.48 (1.11-1.98)  p=0.007 | 2.80 (1.24-6.32)  p=0.013 | 2.94 (0.86-10.14)  p=0.087 | 1.77 (1.17-2.67)  p=0.006 |
| **Allocated treatment** |  |  |  |  |  |
| Con-64Gy | 1.00 (reference) | 1.00 (reference) | 1.00 (reference) | 1.00 (reference) | 1.00 (reference) |
| Esc-74Gy | 0.81 (0.61-1.10)  p=0.176 | 0.76 (0.57-1.01)  p=0.061 | 1.06 (0.55-2.04)  p=0.860 | 1.02 (0.39-2.72)  p=0.953 | 0.82 (0.57-1.19)  p=0.300 |
| **Is patient included in the PP biopsy group** |  |  |  |  |  |
| No | 1.00 (reference) | 1.00 (reference) | 1.00 (reference) | 1.00 (reference) | 1.00 (reference) |
| Yes | 1.16 (0.85-1.58)  p=0.351 | - 1. (0.74-1.36)   p=0.972 | 1.05 (0.53-2.06)  p=0.893 | 0.62 (0.20-1.91)  p=0.410 | 0.63 (0.40-0.97)  p=0.035 |

* timed from two-year PSA value

^¥^ HR comparing “positive” vs “negative or suspicious” biopsy outcome

**Supplementary Table 5: comparison of metastases or death from prostate cancer and overall mortality between the biopsied and non-biopsied groups**

| **2-year biopsy status** |  | **Metastases or Death from prostate cancer** | | **Death from prostate cancer** | | **Death from any cause** | |
| --- | --- | --- | --- | --- | --- | --- | --- |
|  |  | **N** | **%** | **N** | **%** | **N** | **%** |
| 2-year biopsy **performed**  (N=312) | No PSA/Clinical failure at 2 years | 14/242 | (6%) | 6/242 | (2%) | 37/242 | (15%) |
|  | PSA/Clinical failure at 2 years* | 23/70 | (33%) | 17/70 | (24%) | 27/70 | (39%) |
| 2-year biopsy **not performed**  (N=531) | No PSA/Clinical failure at 2 years | 37/448 | (8%) | 19/448 | (4%) | 114/448 | (25%) |
|  | PSA/Clinical failure at 2 years | 58/83 | (70%) | 49/83 | (59%) | 58/83 | (70%) |

*65/70 had bPFS event before the 2yr biopsy, 6^¥^/70 had biopsy too early/late, and for 1^$^/70 neither local nor central biopsy histopathology report was available (see Figure 1)

^¥, $^ one patient satisfies both criteria

**Supplementary Figure 1: Outcome measures, LBG(local histopathology review)**

(A) biochemical Progression-Free Survival, by biopsy outcome biopsy

(B) biochemical Progression-Free Survival, negative-and-suspicious vs positive biopsy

(C) Metastases-Free Survival, by biopsy outcome biopsy

(D) Metastases-Free Survival, negative-and-suspicious vs positive biopsy

(E) Overall Survival, by biopsy outcome biopsy

(F) Overall Survival, negative-and-suspicious vs positive biopsy

| **** | **** |
| --- | --- |
| **** | **** |
| **** | **** |
